# Dendritic Cells Control CD4^+^CD25^+^ Treg Cell Suppressor Function *In Vitro* through Juxtacrine Delivery of IL-2

**DOI:** 10.1371/journal.pone.0043609

**Published:** 2012-09-12

**Authors:** Katarina Kulhankova, Todd Rouse, Mohamed E. Nasr, Elizabeth H. Field

**Affiliations:** 1 Department of Veterans Affairs Medical Center, Iowa City, Iowa, United States of America; 2 Department of Medicine, Roy J. and Lucille A. Carver College of Medicine, The University of Iowa, Iowa City, Iowa, United States of America; New York University, United States of America

## Abstract

CD4^+^CD25^+^Foxp3^+^ regulatory T cells (Tregs) restrict inflammatory responses to self and nonself. Aberrant Treg activity is pathologic: Insufficient Treg activity is implicated in autoimmunity, allergy, and graft-versus-host-disease; overabundant activity is implicated in chronic infection and cancer. Tregs require IL-2 for their expansion and acquisition/execution of suppressor function; however, because Tregs cannot produce IL-2, they depend on IL-2 from an exogenous source. Until now, that IL-2 source had not been established. We asked whether dendritic cells (DCs) could supply IL-2 to Tregs and, if so, what was required for that delivery. We used flow cytometry, IL-2 ELISPOT, RT-qPCR, and IL-2 promoter-driven reporter assays to measure intracytoplasmic IL-2, secreted protein, IL-2 message and IL-2 promoter activity in bone marrow-derived (BMDC) and splenic DCs. We examined conjugate formation between Tregs, conventional CD4^+^ cells, and IL-2-expressing DCs. We measured Treg levels of CD25, Foxp3, and suppressor function after co-culture with IL-2 sufficient and IL-2^−/−^ DCs. We generated IL-2-mCherry-expressing DCs and used epifluorescence microscopy and flow cytometry to track IL-2 transfer to Tregs and test requirements for transfer. Between 0.7 to 2.4% of DCs constitutively produced IL-2 and diverted IL-2 secretion to Tregs by preferentially forming conjugates with them. Uptake of DC IL-2 by Tregs required cell-cell contact and CD25. Tregs increased levels of CD25 and Foxp3 from baseline and showed greater suppressor function when co-cultured with IL-2-sufficient DCs, but not when co-cultured with IL-2^−/−^ DCs. Exogenous IL-2, added in excess of 500 U/ml to co-cultures with IL-2^−/−^ DCs, restored Treg suppressor function. These data support a model of juxtacrine delivery of IL-2 from DCs to Tregs and suggest that a subset of DCs modulates Treg function through controlled, spatial delivery of IL-2. Knowledge of how DCs regulate Tregs should be integrated into the design of interventions intended to alter Treg function.

## Introduction

Natural CD4^+^CD25^+^Foxp3^+^ T regulatory cells (Tregs) comprise only about 1–10% of the pool of CD4^+^ cells, but because they develop and maintain peripheral tolerance to autoantigens, neo-antigens, and foreign antigens [1], [2] they are the primary cells responsible for limiting inflammatory adaptive immune responses. Moreover, their power can extend even to curtailing immunity to pathogens [3] and tumor antigens [4], [5]. Although therapies aimed at enhancing or preventing Treg function are being explored across medical disciplines, an inability to identify unique requirements for Treg activation has remained a barrier to their use in the management of immunologic disease. To date, agents known to expand and activate Tregs have risked enhancing conventional T cell contamination *ex vivo* [6] and conventional T cell activation *in vivo* [7]. Types of Treg-based therapies underway have included efforts to intensify immune tolerance to the autoantigen in type-1 diabetes patients [8], [9], [10], the donor-HLA antigen in solid organ transplant recipients [11], [12], and the host antigen in hematopoietic cell transplant recipients with graft-versus-host-disease [13]. The approach of such therapies has been to increase Treg activity by infusing *ex vivo* expanded Tregs or by introducing biologics or small-molecule chemical compounds that promote Treg development *in vivo* [14], [15]. In addition to the aforementioned indications, efforts are underway to modify cancer immunotherapy to decrease Treg activity [16], [17], since reports that conventional cancer immunotherapy may expand Tregs and accelerate tumor growth [18], [19], [20]. Collectively, the successful application of all Treg-based therapies will require a thorough knowledge of the mechanisms that control Treg function.

Treg expansion and Treg fitness both contribute to Treg effectiveness in suppressing immune responses. In particular, the cytokine IL-2 is recognized as essential to Treg expansion and survival [21]. However, its role in Treg suppressor function has been less clear [22], in part because it is difficult to uncouple Treg function from Treg survival *in vivo*. The biological effect of IL-2 is mediated through its unique IL-2 receptor. The IL-2 receptor consists of three subunits: the CD25 alpha (α) chain, the CD122 beta (β) chain, and the CD132 common cytokine gamma (γ) chain [23]. IL-2 binding to CD25 initiates the assembly of the high affinity IL-2 receptor signaling complex [24], [25].

Studies of knock-out (KO) mice show that *in vivo* Treg development and peripheral expansion require (i) IL-2 from a Treg-extrinsic source and (ii) an intact IL-2 receptor on Treg cells, suggesting that the formation of a functional IL-2/IL-2Rαβγ quaternary complex is necessary for optimizing Treg fitness. IL-2^−/−^, IL-2Rα^−/−^, or IL-2Rβ^−/−^ KO mice have decreased numbers of natural CD4^+^CD25^+^ Tregs [26], [27], [28], [29] and suffer from autoimmunity [30], [31], [32] or fatal lymphoproliferative disease [29]. Wild-type Tregs, after adoptive transfer to IL-2Rβ^−/−^ KO mice, engraft and undergo normal homeostatic proliferation in peripheral lymph nodes [33] and rescue mice from autoimmunity [34]. In contrast, wild-type Tregs, after adoptive transfer to IL-2^−/−^ KO mice, fail to expand in the periphery and fail to prevent autoimmunity [27]. In spontaneous experimental autoimmune encephalomyelitis (EAE) secondary to Treg dysfunction, the adoptive transfer of CD4^+^ T cells from either wild-type or IL-2^−/−^ KO mice conferred protection from EAE, whereas adoptive transfer of CD4^+^ T cells from IL-2Rα^−/−^ KO mice did not [35]. The enforced expression in the IL-2Rβ^−/−^ KO mice of a transgenic chimeric receptor—composed of the extracellular domain of wild-type IL-2Rβ fused to the cytoplasmic domain of the IL-7Rα—rescued the IL-2Rβ^−/−^ KO mice from autoimmunity. In contrast, the transgenic expression of either the wild-type IL-7R or the chimeric receptor composed of extracytoplasmic domain of IL-7Rα fused to the cytoplasmic domain of IL-2Rβ did not [36]. This failure of Tregs to thrive in the absence of a Treg-extrinsic source of IL-2 or access to the components of the IL-2 receptor that confer high affinity binding of IL-2 indicates that Tregs require an ongoing supply of IL-2 for survival.

Similarly, the *in vivo* treatment of mice with either an antibody to neutralize IL-2 or anti-CD25 triggers autoimmune disease [30], [31], [32]. The short-term neutralization of circulating IL-2 by anti-IL-2 monoclonal antibody reduces the number of Tregs in the periphery and elicits autoimmune gastritis in BALB/c mice and diabetes and other autoimmune manifestations in non-obese diabetic (NOD) mice [37]. Furthermore, administration of a relatively “lower” dose of IL-2 (complexed with anti-IL-2) promotes survival of Tregs within islets and retards the development of diabetes in NOD mice [38] and prevents autoimmunity in IL-2^−/−^/Bim^−/−^ double KO mice [39].

Likewise, Treg suppressor function *in vitro* requires that Tregs have intact IL-2 receptors and a Treg-extrinsic supply of IL-2. Tregs suppress proliferation of CD4^+^CD25^−^ cells as well as their transcription and secretion of IL-2; this Treg suppressor function is blocked by antibody against CD25 [40] or a combination of antibodies against CD25 and CD122 [40], [41]. Tregs that are pre-activated and treated with supplemental IL-2 show 20-fold greater capacity for *in vitro* suppression compared with Tregs that are pre-activated in the absence of IL-2 [40], [41]. Moreover, though treatment of Tregs with IL-2, IL-7, or IL-15 triggered STAT5 phosphorylation and increased forkhead box protein 3 (Foxp3) in Tregs, only treatment with IL-2 conferred potent suppressor function to Tregs [42].

Taken together these data favor a model whereby Treg suppressor function depends on IL-2 driving the formation of high affinity IL-2R on the Treg cell. Tregs constitutively express all three chains of the IL-2R [27], [43]. However, because Tregs do not produce IL-2 [28], [40] their suppressor function is controlled through IL-2 supplied by a nearby cell [22], [36].

Until now the identity of that IL-2 source has not been conclusively verified. The activated non-Treg CD4^+^ cell has been touted as a logical candidate IL-2 [37]. This possibility is supported by the fact that *in vivo* development of Tregs occurs when the IL-2 source is restricted to other non-Treg CD4^+^ cells [44]. However, other studies have put forth the possibility that the IL-2 source may be a non-T cell [45] such as the dendritic cell [46], [47].

The aim of our *in vitro* experiments was to determine whether dendritic cells supply IL-2 to Tregs and, if so, the mechanism of that delivery. We demonstrate here that DCs cells are a bona fide source of IL-2 for Tregs, even in the absence of an inflammatory microenvironment. Tregs lose the ability to suppress wild-type CD4^+^CD25^−^ cells (Teff) when IL-2 from DCs is restricted, either by blocking with anti-CD25 or by substituting DCs from IL-2^−/−^ KO mice. The dendritic cells favor close interaction with Treg and preferentially transfer their IL-2 to Treg via a cell-cell, contact-dependent juxtacrine process. Thus, we identify for the first time the existence of a unique subset of IL-2-producing dendritic cells that provide Tregs with a supply of critical IL-2 needed to gain suppressor function. Furthermore, we show for the first time that dendritic cell IL-2 regulates Treg Foxp3 concentration and suppression function *in vitro*. These results provide insights that should help inform the design of interventions aimed at enhancing or inhibiting Treg function.

## Results

### Small subpopulation of dendritic cells constitutively produces IL-2

**Figure 1:**
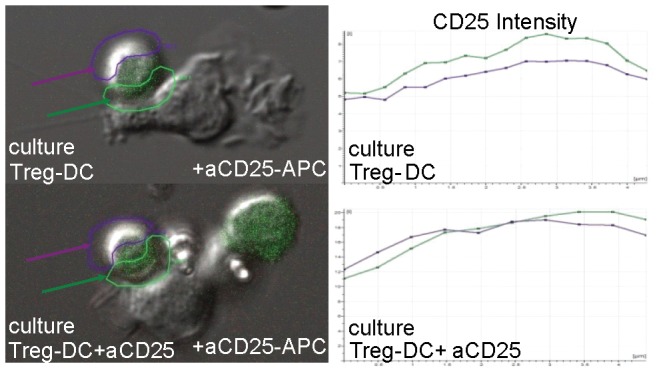
CD25 localizes to Treg:dendritic cell contact site. Foxp3-GFP Treg cells from C.CgFoxp3tm2Tch/J mice were co-cultured with BALB/cJ splenic DCs and anti-CD3 with or without unlabeled anti-CD25 antibody. Live cell confocal imaging was performed on Treg:DC conjugates 24 h later as in materials and methods. Anti-CD25-APC was added to the cultures prior to collecting imaging data. **Left**: DIC/GFP/APC projection images of Treg:DC conjugate from culture without blocking, unlabeled anti-CD25 (upper) and culture with blocking, unlabeled anti-CD25 (lower). **Right:** The corresponding LCS-Lite analysis of the relative intensity of anti-CD25-APC (y-axis) on Treg membrane not contacting (purple) or in contact with (green) the DC across multiple z-stacks (x-axis).

**Figure 2:**
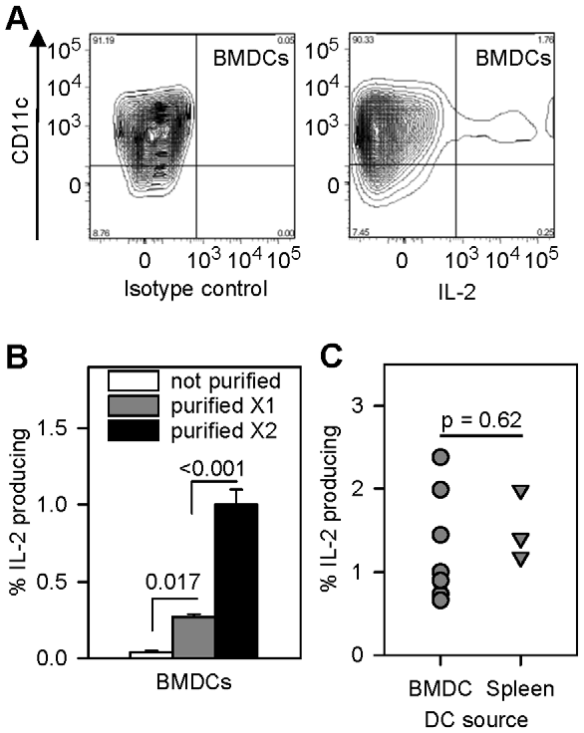
A small subpopulation of dendritic cells secrete IL-2 protein. **A:** CD11c^+^ dendritic cells contain IL-2 protein. DCs were stained for intracytoplasmic IL-2 as in materials and methods. Panels show FACS contour plots of BMDCs stained with anti-IL-2 (right) or isotype control antibody (left). The experiments were repeated with similar results. **B:** Progressive purification of CD11c^+^ dendritic cells enriches for IL-2 producing cells. The frequency of spontaneous IL-2 secreting BMDCs was measured by IL-2 ELISPOT assay as in materials and methods. Bars show mean ± SE of % IL-2-producing cells in triplicate samples of unpurified, 1X and 2X MACS-purified CD11c^+^ BMDCs. Statistical analysis was by one-way repeated measures ANOVA and Tukey's test for all pairwise multiple comparison procedure. **C:** Dendritic cells were doubly-purified and the percentage of IL-2-producing bone marrow-derived DCs ranged from 0.7 to 2.4% (N = 7), and the percentage of IL-2-producing splenic-derived DCs ranged from 1.2 to 2.0 (N = 3).

The observation from live-cell imaging studies that CD25 localized at the interface between some Treg-DC conjugates (Figure 1) prompted our investigation of DCs as a source of IL-2 to Tregs. We examined bone marrow-derived dendritic cells (BMDCs) and splenic DCs for their capacity to produce IL-2 constitutively. We first confirmed that splenic DCs and bone marrow-derived BMDCs transcribed IL-2 mRNA (not shown), using reverse transcription quantitative real-time polymerase chain reaction (RT-qPCR). Figure 2A demonstrates by flow cytometry that 1.9% of gated CD11c^+^ BMDCs contained intracytoplasmic IL-2 protein. Likewise, we detected intracytoplasmic IL-2 protein in a similarly small subpopulation of splenic DCs (not shown). Using IL-2 ELISPOT assay and progressively purified DCs, we confirmed that a subset of DCs secreted IL-2 (Figure 2B). Progressive enrichment of CD11c^+^ cells (61% to 98% after two rounds of magnetic bead selection), increased the percentage of IL-2-secreting DCs significantly (0.04 to 1% of the DC population). In a series of experiments performed over several months on doubly-selected BMDCs or splenic DCs and shown in Figure 2C, the percent of IL-2-producing BMDCs ranged from 0.7 to 2.4% (mean was 1.3%±0.7, N = 7), and the percent of IL-2 producing, splenic DCs ranged from 1.2 to 2.0% (mean was 1.5%±0.4, N = 3). There was no significant difference between the percentages of IL-2 producing cells from the two sources of dendritic cells in Figure 2C (p = 0.62, t-test).

### Tregs preserve dendritic cell transcription of IL-2

**Figure 3:**
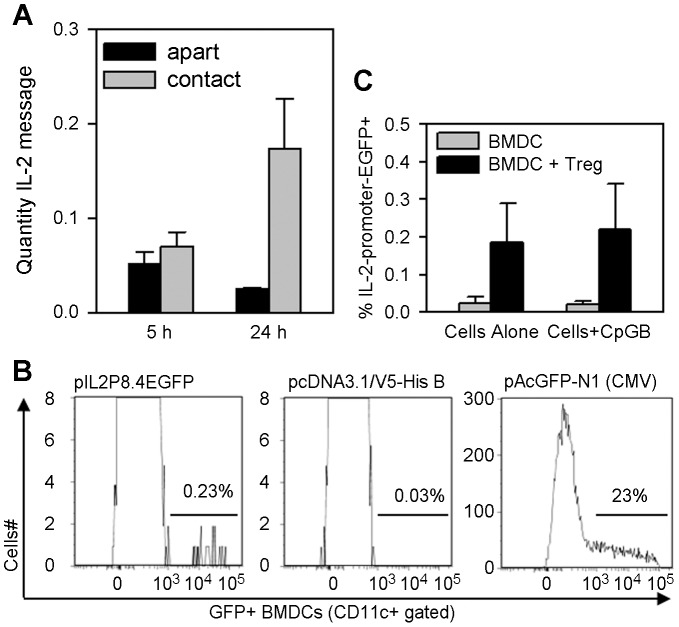
Tregs preserve dendritic cell transcription of IL-2. **A:** DO11.10 Tregs and C57BL/6 splenic DCs were cultured together or in separate tubes for 5 and 24 h, IL-2 message was measured in cultured cells by RT-qPCR as in materials and methods. Bars are the mean ± SE of IL-2 message in the cultures. Statistical analysis was by one-way repeated measures ANOVA and Tukey's test for all pairwise multiple comparison procedure. **B&C:** BMDCs were transfected with the IL-2 reporter plasmid (pIL2P8.4-EGFP), a negative control plasmid (pcDNA3.1/V5-His B), and a positive control plasmid to assess transfection efficiency (pAcGFP-N1). Transfectants were analyzed for IL-2-promoter activity in cells as in materials and methods. **B:** Panels show histograms of the level of promoter-driven GFP in gated CD11c^+^ cells. The experiment was repeated with similar results. **C:** pIL-2P8.4-EGFP-transfected BMDCs were cultured unstimulated or with CpG-B, with and without Treg cells for 40 h. Bars are the mean ± SE of IL-2-promter-EGFP^+^ BMDCs from two experiments.

We then cultured C57BL/6 DCs alone or with alloreactive Tregs from the DO11.10 mouse and measured IL-2 message by RT-qPCR. Figure 3A shows that IL-2 message waned as DCs were cultured alone over time but was significantly higher in cells that were co-cultured with Tregs (p = 0.038, Tukey Test). To corroborate these observations and further confirm that IL-2 was coming from the DC, we transfected BMDCs with an IL-2 promoter-driven reporter plasmid, pIL2p8.4EGFP (Figure 3B, left panel), which uses the 8.4 kb IL-2 promoter fragment to drive enhanced green fluorescent protein (EGFP) transcription. This method allowed us to use flow cytometry to detect individual DCs with endogenous IL-2 promoter activity. After adjusting for the 23% transfection efficiency of the cells (see Figure 3B, right panel), Figure 3B, left panel shows approximately 1% of CD11c^+^ BMDCs with endogenous IL-2 promoter activity, within the range of BMDCs that secreted IL-2 protein in ELISPOT assays in Figure 2C. Figure 3C shows a higher percentage of DCs with endogenous IL-2 promoter activity when DCs were co-cultured with Tregs compared to DCs cultured alone. Thus, by four separate readouts—IL-2 promoter activity, message, intracellular protein, and secreted protein—we conclude that a small subpopulation of DCs constitutively makes IL-2 protein. Moreover, interaction with Treg may be necessary to sustain IL-2 producing DCs.

### IL-2-producing dendritic cells form conjugates with Treg cells

We performed flow cytometric analysis of BMDCs and splenic DCs from C57Bl/6-Tg-IL2p8-4 transgenic mice (gift from Ellen Rothenberg, California Institute of Technology) to define markers for the DC subset with IL-2 promoter activity. These studies showed that the subset was CD11c^+^/CD4^−^/CD8^−^/CD25^−^/CD103^−^/CD3^−^/Siglec-H^−^/CD205^−^, indicating that IL-2-transcribing DCs belong to a non-plasmacytoid, non-CD8α DC population.

**Figure 4:**
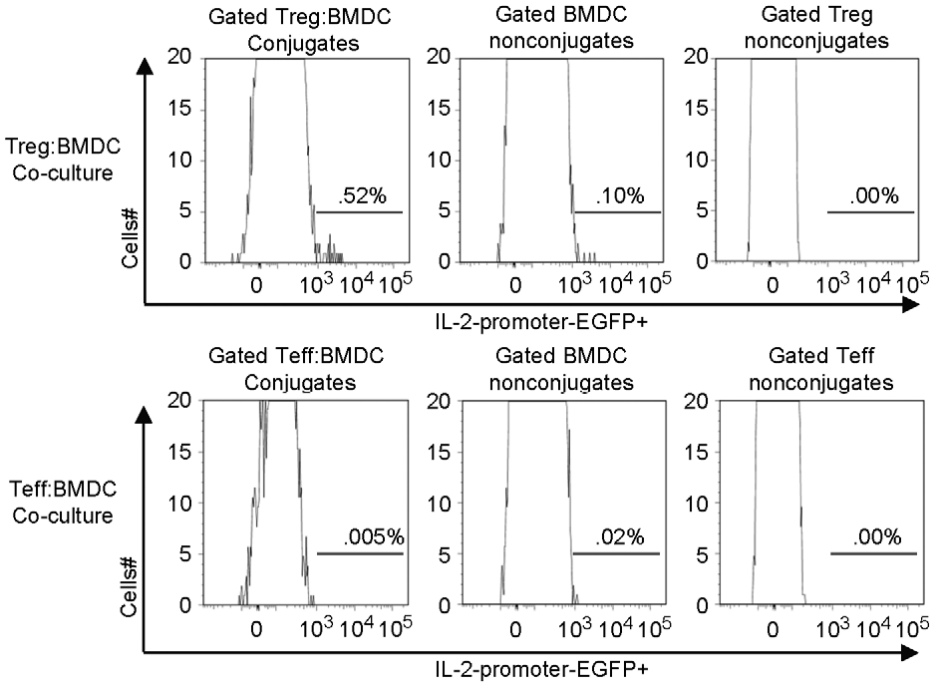
IL-2-producing dendritic cells form conjugates with Treg cells. BMDCs were transfected with IL-2 reporter plasmid (pIL2P8.4-EGFP) or control plasmids and analyzed for IL-2-promoter activity in cells as in materials and methods. pIL-2P8.4-EGFP-transfected C57BL/6 BMDCs were cultured with DO11.10 Treg cells or DO11.10 CD4^+^CD25^−^ Teff cells for 40 h. Panels show histograms of the level of IL-2-promoter-EGFP^+^ activity in gated populations: DCs conjugated with Treg or Teff cells, and non-conjugated cells.

In the absence of unique subset markers we relied on DCs that were transfected with the IL-2-reporter plasmid pIL2p8.4EGFP to track the behavior of DCs with IL-2 promoter activity. We then examined IL-2 promoter activity of C57BL/6 DCs that were non-conjugated or conjugated with alloreactive DO11.10 CD4^+^CD25^+^ Tregs or CD4^+^ Teff cells. Figure 4 shows an enrichment of DCs with IL-2 promoter activity in the Treg-DC conjugates. One interpretation of these data is that IL-2-producing DCs interact preferentially with Tregs in culture.

### Tregs intercept dendritic cell IL-2

**Figure 5:**
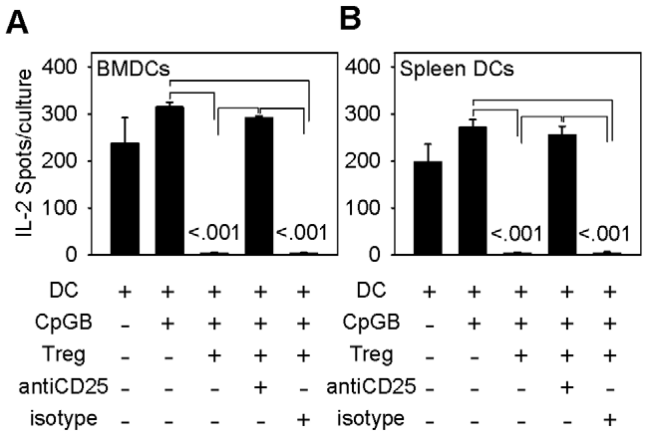
Tregs interfere with dendritic cell paracrine secretion of IL-2. Treg interference of dendritic cell paracrine secretion of IL-2 depends on CD25. C57BL/6 splenic DCs (left) and BMDCs (right) were cultured in IL-2 ELISPOT plates unstimulated or with CpG-B, with and without DO11.10 Treg cells, anti-CD25, or isotype control, and the number of IL-2 secreting DCs was measured as in materials and methods. Bars are the mean ± SE of IL-2 secreting DCs in triplicate samples of one of six representative experiments with splenic DCs (left) and one of three representative experiments with BMDCs (right). Statistical analysis by one-way repeated measures ANOVA and Tukey's test for all pairwise multiple comparison procedure.

Data from Figure 4 also suggests that IL-2 promoter activity is preserved in DCs in conjugate formation with Tregs. We then examined the effect of Tregs on IL-2 protein secretion by the DCs. We cultured C57BL/6 DCs with and without alloreactive DO11.10 Tregs in IL-2 ELISPOT plates and measured the number of IL-2-producing DCs. In addition, since it has been reported that DCs produce IL-2 in response to toll-like receptor (TLR) signals [48], [49], [50] we examined the effect of different TLR agonists on the DCs in our system. About 2% of freshly isolated splenic DCs (Figure 5, left panel) and BMDCs (Figure 5, right panel) secreted IL-2 constitutively. Treatment with the TLR9 agonist CpG-B increased the number of IL-2-secreting DCs (Figure 5), whereas treatment with the TLR4 agonist LPS (lipopolysaccharide) did not alter the number of IL-2-secreting DCs (not shown).

Tregs profoundly abrogated detection of IL-2-secreted protein by DC. We did not detect secreted IL-2 in more than a dozen experiments where Tregs were co-cultured with DCs in IL-2 ELISPOT, regardless of whether we measured constitutive, CpG-B-induced, or LPS-induced IL-2 responses (Figure 5 and data not shown). Because IL-2 promoter activity was preserved in DCs that were conjugated to Tregs (Figure 4), and because CD25 localized to the interface between Treg-DC conjugates (Figure 1), we reasoned that DCs secrete IL-2 directly to adjacent Tregs (Figure 5). In further support of this interpretation, we demonstrated that the addition of anti-CD25 to co-cultures of Tregs and DCs completely restored the detection of IL-2-secreting DCs in IL-2 ELISPOT, whereas the addition of isotype control antibody had no effect (Figure 5). Thus, in the absence of Tregs, ELISPOT plates capture the IL-2 that DCs secrete. In the presence of Tregs, via their CD25 surface receptor, Tregs capture the IL-2 that DCs secrete.

### Tregs require CD25 and cell-cell contact with dendritic cells to take up IL-2

**Figure 6:**
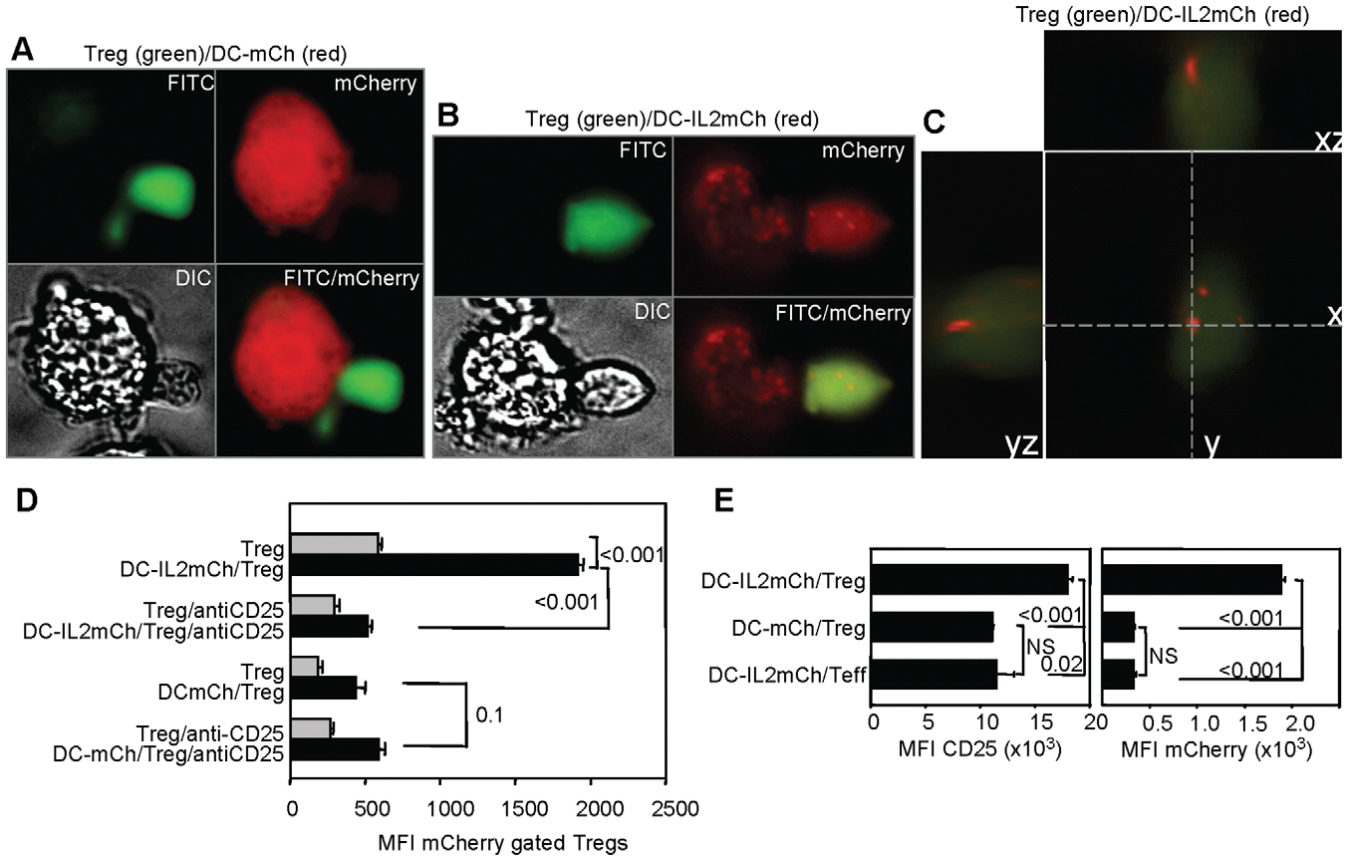
CD25 and cell contact dependent uptake of dendritic cell IL-2 by Treg cells. **A–C:** CFSE-labeled DO11.10 Tregs were stimulated with OVA peptide and different DC transfectants as in materials and methods. Panels show representative images of mCherry transfected dendritic cell (A) and IL-2mCherry transfected dendritic cell (B) in conjugate formation with Treg cell. Panels C: Three-dimensional rendering of a Treg cell localizes the IL-2mCherry to vesicles within Treg. **D&E:** DC-Treg contact facilitates IL-2mCherry transfer from DCs to Tregs that can be blocked by anti-CD25. DO11.10 CD4^+^CD25^+^ Treg or CD4^+^CD25^−^ T effector (Teff) cells were cultured in triplicate in upper chambers of transwells separated from (grey bars) or in lower chambers of transwells in contact with (black bars) BMDCs from IL-2^−/−^ B6 mice that were transfected with IL-2mCherry (DC-IL2mCh) or control mCherry (DCmCh) and analyzed by flow cytometry as in materials and methods. Indicated cultures contained anti-CD25 antibody (antiCD25). **D:** Bars are the mCherry MFI means ± SE of gated Thy1.2high cells from triplicate lower chambers (black) and upper chambers (gray). **E:** Bars are the CD25 (left panel) and mCherry (right panel) MFI means ± SE of gated Thy1.2high cells from triplicate lower chambers (black). Statistical analysis was performed by paired *t*-test for within well samples and by student t-test for between well samples. Experiment was performed three times yielding similar results, representative experiments are shown.

Utilizing two-photon laser-scanning microscopy to characterize Treg behavior *in vivo* during development of autoimmune priming in diabetes-prone mice, Tang et. al, reported that Treg cells in pancreatic draining lymph node form stable conjugates with dendritic cells but not with CD4^+^ Teff cells [51]. Therefore, we tested whether DCs formed conjugates with Tregs to allow IL-2 transfer. We transfected BMDCs with plasmids encoding IL-2-mCherry fusion protein (or control mCherry), and we performed multi-color live cell imaging with epifluorescence microscopy to track IL-2 delivery from DCs. We first examined cultures of DO11.10 Tregs that were stimulated with OVA peptide and BALB/c transfected BMDCs. DCs that were transfected with mCherry displayed the mCherry throughout their cytoplasm, whereas DCs that were transfected with IL-2-mCherry fusion protein displayed the IL-2-mCherry in intracellular vesicles (compare mCherry distribution in Figures 6A and 6B). Figure 6B shows that IL-2-mCherry was transferred to a Treg cell conjugated with an IL-2-mCherry-expressing DC. Figure 6C illustrates that the IL-2-mCherry was located within the Treg cell, indicating that IL-2 is endocytosed by the Treg and not merely on the cell surface. However, we also detected some IL-2-mCherry Treg cells that were not conjugated to DCs (not shown). Because the images represent a snap shot of the co-culture after 24 hours, we were not able to determine whether the later mCherry Treg cells were ever conjugated to IL-2-producing DCs.

To explore the question of whether DCs transferred IL-2 to Tregs via direct cell contact we made use of the transwell system. We transfected IL-2^−/−^ BMDCs with plasmids encoding IL-2-mCherry fusion protein or mCherry, and we cultured the transfected cells in the lower chamber of transwell plates. Alloreactive DO11.10 Tregs were labeled with carboxylfluorescein succinimide ester (CFSE) and added to both lower and upper chambers. Flow cytometry was performed on the Treg from upper and lower chambers. The Tregs that were co-cultured in contact with IL-2-mCherry-producing DCs in the same lower transwell chamber (Figure 6D, black bars) took up significantly more IL-2-mCherry than Tregs that were cultured in the upper chamber separately from the DCs (Figure 6D, gray bars; compare top pair of gray and black bars, *p*<.001). The addition of anti-CD25 to the transwell plate blocked uptake of IL-2-mCherry by Tregs co-cultured in the lower chamber in contact with IL-2-mCherry-producing DCs to the background level (Figure D, *p*<.001). These results indicate that cell-cell contact and CD25 are required for efficient transfer of IL-2 from the DC to the Treg cell.

Compared to Tregs, there was no transfer of IL-2-mCherry to CD4^+^ Teff cells that were co-cultured in lower chambers in contact with IL-2-mCherry-producing DCs (Figure 6E right panel, top vs. bottom bars, *p*<.001). This was not from lack of CD25 expression on CD4^+^ Teff cells. The level of CD25 on the stimulated CD4^+^ Teff cells in contact with IL-2-mCherry-producing DCs was the same as the level of CD25 on the Treg cells in contact with control, mCherry-expressing DCs (Figure 6E left panel, middle vs. bottom bars, not significant). These data suggest that IL-2-producing DCs preferentially form conjugates with Tregs to facilitate juxtacrine delivery of IL-2.

### Dendritic cell IL-2 increases Treg expression of CD25 and Foxp3

**Figure 7:**
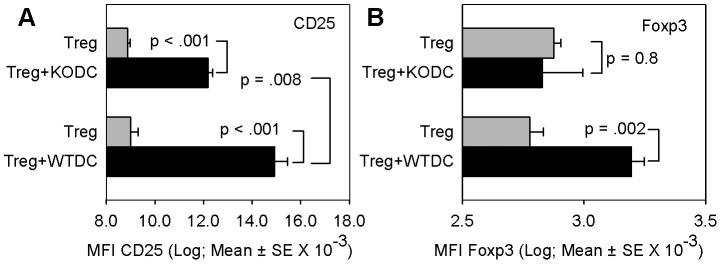
Dendritic cell IL-2 increases Treg phenotype. DO11.10 CD4^+^CD25^+^ Treg cells were cultured without or with BMDCs from either WT or IL-2^−/−^ KO (on B6 background) mice in transwells and the level of CD25 (A) and Foxp3 (B) on Treg cells was measured by flow cytometry as in materials and methods. **A:** Bars are the mean ± SE of mean fluorescent intensity (MFI) of CD25 levels on Thy1.2-gated Tregs of triplicate samples from one of four representative experiments. Statistical analysis was by Tukey's test for all pairwise comparisons. **B:** Bars are the mean ± SE of mean fluorescent intensity (MFI) of Foxp3 levels on Thy1.2-gated Tregs of triplicate samples from a single experiment. Statistical analysis was by Tukey's test for all pairwise comparisons.

IL-2 increases expression of CD25 on CD4^+^ cells [52] and Foxp3 in Tregs [29], [42], [53]. We noted that Tregs in contact with IL-2-mCherry-producing DCs that took up IL-2mCherry expressed higher levels of CD25 than Treg cells in contact with control, mCherry-expressing DCs (Figure 6E left panel, top vs. middle bars, *p*<.001). To further examine whether DC-secreted IL-2 affects Treg phenotype we compared the baseline level of CD25 and Foxp3 protein in DO11.10 Tregs that had been cultured alone with the levels of CD25 and Foxp3 protein in Tregs cultured with either wild-type or IL-2^−/−^ KO BMDCs. Figure 7A shows that Tregs co-cultured with wild-type DCs expressed significantly higher levels of CD25 compared with Tregs cultured alone (p = <.001, Tukey's Test) or with DCs from IL-2^−/−^ mice (p = .008, Tukey's Test). Figure 7B shows that Tregs co-cultured with wild-type DCs expressed significantly higher levels of Foxp3 compared with Tregs cultured alone (p = .002, Tukey's Test). In contrast, there was no difference in the level of expression of Foxp3 in Tregs co-cultured with DCs from IL-2^−/−^ mice compared with Tregs cultured alone (p = 0.8, Tukey's Test). These data indicate a biological response to IL-2 by Tregs in culture with IL-2-sufficient DCs and provide further evidence that DCs can supply IL-2 to Tregs.

### Dendritic cell IL-2 is essential for *in vitro* suppressor function of CD4^+^CD25^+^ Tregs

**Figure 8:**
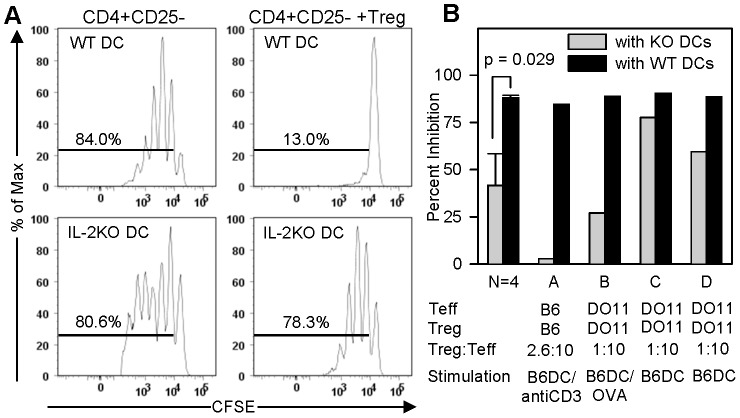
Dendritic cell IL-2 regulates Treg suppression of CD4+ proliferation. **A:** The Panels show proliferation of anti-CD3 stimulated CFSE-labeled C57BL/6 CD4^+^CD25^−^ Teff cells co-cultured with WT or IL-2^−/−^ KO (on B6 background) BMDCs, with and without C57BL/6 Treg cells at a Treg:Teff ratio of 2.5∶10. Numbers indicate percentage of Teffs that have divided. **B:** CD4^+^CD25^−^ Teff cell proliferation was measured by flow cytometry, and the % of Treg suppression of Teff cell proliferation was calculated as in materials and methods. Bars show percent Treg inhibition of proliferation of CD4^+^CD25^−^ Teff cells that were co-cultured with IL-2^−/−^ KO (gray) or WT (black) B6 BMDCs and Tregs at Treg:Teff ratio of 2.5∶10 (A) or 1∶10 (B–D), and stimulated as specified in columns A–D and materials and methods.

The suppressor function of mouse Tregs has been linked to their level of Foxp3. The finding that DC-secreted IL-2 increases Treg expression of Foxp3 (Figure 7B) raised the possibility that DC-secreted IL-2 imparts Treg suppressor function. We directly examined the effect of DC-supplied IL-2 on Treg function by conducting Treg suppressor assays with either DCs from wild-type or IL-2^−/−^ mice. We utilized two kinds of Treg suppressor assays. The first type was the standard Treg suppressor assay, which measures Treg inhibition of proliferation of polyclonally-activated CFSE-labeled conventional CD4^+^ Teff cells. CD4^+^ Teff cells proliferate to a similar degree when stimulated with anti-CD3 in the presence of syngeneic DCs from either wild-type or IL-2^−/−^ mice (84% versus 81% of cells proliferated, respectively, Figure 8A and Figure 8B, column A). The addition of syngeneic Tregs to cultures that contain wild-type DCs decreased proliferation of CD4^+^ Teff cells from 84% to 13% (85% suppression). In contrast, Tregs were minimally suppressive (3% suppression) when added to cultures that contained DCs from IL-2^−/−^ mice. These data indicate that IL-2 from DCs is required for Treg suppressor function, but not for CD3-induced proliferation of CD4^+^ Teff cells. We performed the Treg suppressor assays in systems using DO11.10 Treg cells stimulated with wild-type or IL-2^−/−^ DCs plus OVA peptide (Figure 8B, column B) and DO11.10 Treg stimulated with wild-type or IL-2^−/−^ DCs (Figure 8B, columns C and D). Across different stimulation protocols, IL-2 KO DCs rendered Tregs less suppressive of CD4+ Teff cell proliferation (42% versus 88%, p = .029, Mann-Whitney Rank Sum Test). We were unable to test the effect of anti-CD25 or exogenous IL-2 on Treg suppressor function in this system because anti-CD25 and exogenous IL-2 alter baseline proliferation of CD3-stimulated CD4^+^ Teff cells.

Utilizing the second type of Treg suppressor assay, we tested Treg-mediated suppression of the late-phase of IL-2 production by CD4^+^ Teff cells. Though Tregs do not inhibit the early phase of IL-2 production by activated CD4^+^ Teff cells, Tregs have been shown to truncate IL-2 transcription and suppress the late-phase of IL-2 production by activated CD4^+^ Teff cells [54], [55]. One advantage of the second suppressor assay is that, because IL-2 production by CD4^+^ Teff cells does not depend on exogenous IL-2, we were able to test the effects of both anti-CD25 and exogenous IL-2 on Tregs in our system.

**Figure 9:**
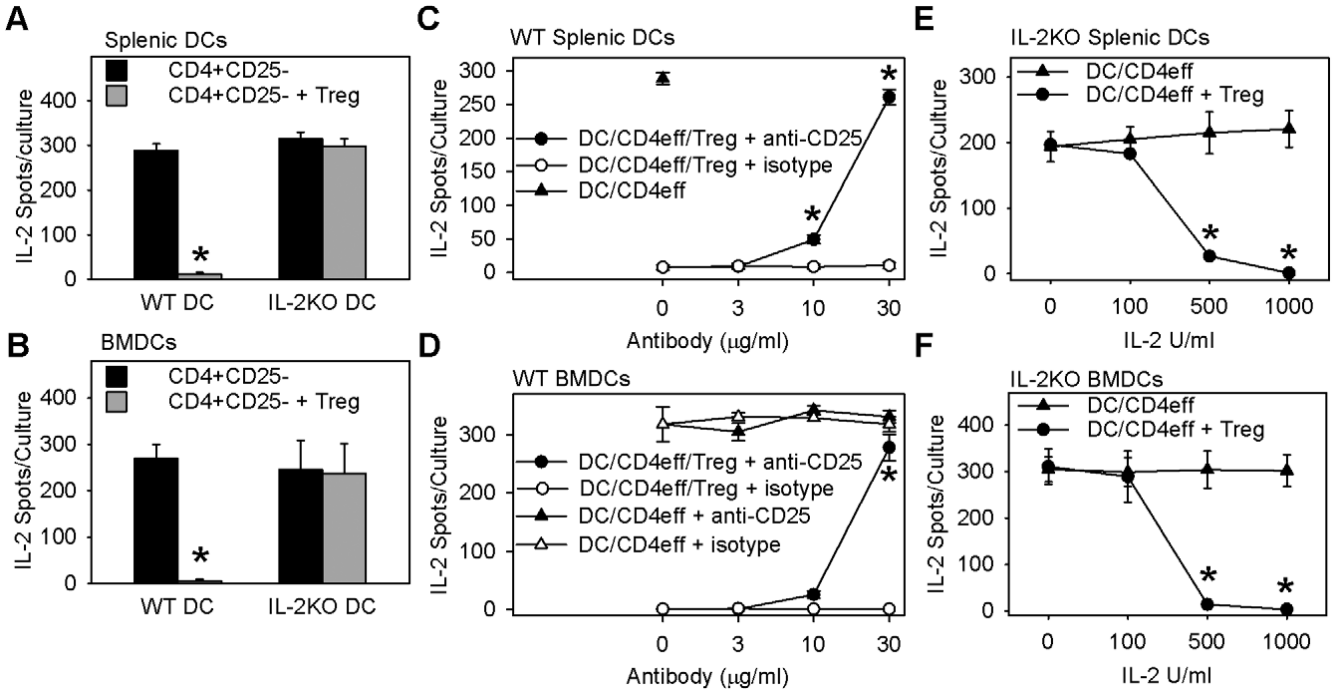
Treg suppression of the MLR-IL-2 response requires dendritic cell IL-2. Panels A–F show Treg suppression of IL-2-secreting cells in primary MLR cultures. MLR cultures of DO11.10 CD4^+^CD25^−^ cells and allogeneic DCs from WT or IL-2^−/−^ KO littermates were incubated overnight with or without DO11.10 Tregs, washed, and transferred to IL-2-ELISPOT plates to measure IL-2-secreting cells as per materials and methods. **A&B:** Tregs fail to suppress MLR-IL-2 response in cultures containing IL-2^−/−^ KO DCs. Bars are mean ± SE IL-2 producing cells/culture from N = 5 independent experiments with splenic DCs (A) and N = 3–4 independent experiments with BMDCs (B). * indicates *p*<.001 (A) and *p*<.005 (B) compared with all other conditions, Holm-Sidak method of multiple pairwise comparison. **C&D:** Anti-CD25 blocks Treg suppression of MLR-IL-2 response. MLR cultures of DO11.10 CD4^+^CD25^−^ cells and allogeneic WT splenic DCs (C) or BMDCs (D), with or without Treg, were treated with anti-CD25 or isotype control antibody, washed, then transferred to IL-2-ELISPOT plates as in material and methods. C: Symbols are mean ± SE IL-2 producing cells/culture from N = 6 independent experiments. * indicates *p*<.001, anti-CD25 versus isotype control, Holm-Sidak method of multiple pairwise comparison. D: Symbols are mean ± SE IL-2-producing cells of triplicate cultures from one of four representative experiments. * indicates *p*<.001, Treg treatment groups anti-CD25 versus isotype control, Tukey's test for all pairwise multiple comparison. **E&F:** Exogenous IL-2 restores Treg suppression of MLR cultures containing IL-2KO DCs. MLR cultures of DO11.10 CD4^+^CD25^−^ cells and allogeneic IL-2^−/−^ KO splenic DCs (E) or BMDCs (F), with or without Treg, were treated with exogenous IL-2, washed, then transferred to IL-2 ELISPOT plates as in material and methods. Symbols are mean ± SE IL-2 producing cells of triplicate cultures from one of three representative experiments. * indicates *p*<.001, IL-2 treatment DC/CD4eff + Treg versus DC/CD4eff control, Tukey's test for all pairwise multiple comparison.

DO11.10 Tregs were added to mixed lymphocyte cultures containing DO11.10 CD4^+^ Teff cells (responder cells) and allogeneic BMDCs or splenic DCs (stimulator cells) from B6 mice, and the ability of Tregs to suppress the late phase of IL-2 secretion by DO11.10 CD4^+^ Teff cells (beyond 24 h) was measured by ELISPOT. CD4^+^ Teff cells showed similar late-phase IL-2 response when stimulated with allogeneic DCs from either wild-type or IL-2^−/−^ B6 mice (Figure 9A&B, compare black bars). Thus, CD4^+^ Teff function is independent of DC IL-2. Tregs suppressed IL-2 secretion by alloreactive DO11.10 CD4^+^ Teff responder cells by more than 95% when added to mixed lymphocyte cultures that contained allogeneic wild-type C57BL/6 DCs. In contrast, Tregs showed no suppression of alloreactive DO11.10 CD4^+^ Teff cells when added to cultures that contained allogeneic IL-2^−/−^ B6 DCs (Figure 9A&B, compare gray bars). These data indicate that *in vitro* Treg suppressor function depends entirely on IL-2 supplied by DCs, but not on IL-2 produced by Teff CD4^+^ cells.

The addition of anti-CD25 to cultures containing alloreactive DO11.10 CD4^+^ Teff cells and wild-type allogeneic DCs did not alter the IL-2 response by CD4^+^ Teff cells (Figure 9D, compare open and closed triangles). In contrast, the addition of anti-CD25 blocked the Treg-mediated suppression of the late-phase IL-2 response by CD4^+^ Teff cells (Figure 9C&D, compare open and closed circles). The addition of exogenous IL-2 to the mixed lymphocyte cultures containing alloreactive DO11.10 CD4^+^ Teff cells and DCs from IL-2^−/−^ (B6 background) mice did not change their late-phase IL-2 response (Figure 9E&F, compare triangles). In contrast, the addition of exogenous IL-2 at doses that exceeded 500 U/ml restored Treg-mediated suppression of CD4^+^ Teff cells' late-phase IL-2 response (Figure 9E&F, compare circles). These data confirm that Tregs require IL-2 from DCs to attain suppressor function. Moreover, in the absence of IL-2-producing DCs, the recovery of Treg suppressor function requires exogenous IL-2 at concentrations higher than 500 U/ml, suggesting that DCs utilize spatial control to target delivery of relatively higher concentrations of IL-2 to Tregs.

## Discussion

Our data demonstrate a mechanism whereby dendritic cells play a vital role in controlling Treg cell suppressor function *in vitro* by supplying essential IL-2 to Tregs. First, we show that a subset of dendritic cells constitutively secrete IL-2. Second, we show Tregs consume DC IL-2, as evident by the increased expression of CD25 and Foxp3 by Tregs cultured with IL-2-sufficient dendritic cells. Third, we show that dendritic cells provide the essential source of IL-2 to Tregs via cell-cell contact and CD25-dependent juxtacrine delivery. Finally, we show that IL-2 from dendritic cells regulates Treg suppression *in vitro*.

### A subset of dendritic cells constitutively secretes IL-2

Freshly isolated mouse splenic and bone marrow-derived dendritic cells constitutively secreted IL-2 protein in IL-2 ELISPOT plates (Figures 2B&C and 5). The IL-2 ELISPOT specifically detected IL-2 protein, since dendritic cells from IL-2 knockout mice produced no IL-2 spots when cultured on IL-2 ELISPOT plates, nor did wild-type dendritic cells produce spots when cultured on plates that were coated with an irrelevant isotype control antibody (not shown). Stimulation of either splenic or bone marrow-derived dendritic cells with CpG-B consistently enhanced IL-2 production (Figure 5). We confirmed that dendritic cells were the source of the IL-2 by intracellular detection of IL-2 protein (Figure 2A) and IL-2 promoter activity (Figure 3B) in CD11c^+^ cells. We observed IL-2 promoter activity in bone marrow-derived CD11c^+^ dendritic cells that were transfected with either the pIL2P8.4EGFP (Figure 3B) or the pIL2P7.4→EYFP (not shown) reporter constructs—both constructs with sense IL-2 promoter fragments driving expression of fluorescent protein in the cell—but not in dendritic cells that were transfected with an anti-sense IL-2 promoter construct (not shown).

To our knowledge ours is the first report of constitutive secretion of IL-2 by splenic dendritic cells and bone marrow-derived DCs. Granucci et al. first reported that dendritic cells produce IL-2. Their gene expression microarray studies of bone marrow-derived dendritic cells revealed upregulated IL-2 message in dendritic cells that were stimulated with gram-negative bacteria [56]. Subsequently Granucci et al. showed that stimulating bone marrow-derived or splenic dendritic cells through CD40 or with various microbial products that signal through TLR 2, 4, or 9, increases IL-2 transcription and secretion [57]. Other investigators have independently confirmed that LPS-matured bone marrow-derived dendritic cells produce IL-2 [46], [58]. Marguti et al. further reported that co-culture with allogeneic apoptotic cells induces IL-2 production by immature dendritic cells and enhances IL-2 production by mature dendritic cells [59]. However, these studies did not report any IL-2 production by unstimulated dendritic cells. An important implication of our finding is that IL-2-producing dendritic cells may control Treg fitness *in vivo* even in the absence of infection or other danger signals.

Our use of the ELISPOT assay, a more sensitive assay than ELISA [60], [61], likely enhanced our ability to detect IL-2-producing dendritic cells in the absence of any stimulation. We did not detect IL-2 in any culture supernatant using the ELISA assay (not shown), whereas we consistently detected IL-2-producing DCs by ELISPOT. We found it necessary to modify the ELISPOT assay to detect IL-2-producing DCs. Culturing the DCs directly in the ELISPOT plates optimized detection of IL-2 spots. No spots were detected if we pre-cultured the DC preparations in tubes overnight and washed the cells before transfer to ELISPOT plates (not shown)—the method used to detect IL-2-producing T cells. Although IL-2-producing DCs were detected, as yet other unique markers for these cells have not been identified.

Conversely, two groups were unable to measure IL-2 message or protein in bone marrow-derived dendritic cells, whether or not DCs were matured with LPS [62], [63]. The inconsistent reports of IL-2 production by dendritic cells may be explained by differences in DC culture conditions. Sauma et al. demonstrated that adding IL-4 during the differentiation of bone marrow-derived dendritic cells inhibits IL-2 secretion by the dendritic cells [58]. Of note, investigators who matured bone marrow-derived dendritic cells with IL-4 did not detect IL-2 [62], [63], whereas investigators who omitted IL-4 during differentiation or maturation detected IL-2 [56], [57], [58], [59], [64]. Similarly, we omitted IL-4 from our bone marrow-derived culture conditions and detected IL-2-producing dendritic cells.

### Tregs consume dendritic cell IL-2

The expansion of Tregs depends on a paracrine source of IL-2 [21]. Reports that dendritic cells trigger Treg cell proliferation *in vitro* in the absence of exogenous IL-2 raise the possibility that dendritic cell IL-2 may control Treg proliferation *in vitro* and Treg expansion and homeostasis *in vivo*. Guiducci et al. showed that Treg proliferation *in vitro* and Treg survival *in vivo* depends on IL-2 secretion from dendritic cells, which is prompted by CD40-CD40L interaction between dendritic cells and Tregs [47]. Suffner et al. reported that adoptively transferred Tregs divided less in DC-depleted recipient mice [65]. Sgouroudis et al. reported higher IL-2 transcription in LPS-matured BMDCs from the diabetes-protective NOD.B6 *Idd3* congenic mouse compared to BMDCs from the diabetes-prone NOD mouse [46]. *In vitro*, Tregs from either disease-prone or disease-resistant mice proliferate more to NOD.B6 *Idd3* BMDCs compared to NOD BMDCs, and anti-IL-2 blocks the proliferation. Moreover, *in vivo* priming with NOD.B6 *Idd3* BMDCs expands Foxp3^+^ Treg cells in the draining pancreatic lymph node of recipient NOD congenic mice [46]. Yamazaki et al. showed that dendritic cells produce IL-2 and expand Treg cells in culture, but they dismissed dendritic cells as the paracrine source of IL-2, finding no difference in Treg proliferation in response to wild-type or IL-2 knockout dendritic cells. They concluded that a contaminating T-cell population was the source of the small amounts of IL-2 measured in the cultures that contained IL-2 knockout dendritic cells [64]. Marguti et al. similarly found that dendritic cells produce IL-2 and expand Tregs in culture. These investigators did not determine the identity of the IL-2 source in their cultures but speculated it to be activated CD4^+^ cells [59]. Brinster and Shevach also reported that dendritic cells induce Treg proliferation. However, unable to detect IL-2-producing dendritic cells, they concluded that DC-driven proliferation of Tregs was primarily independent of IL-2 [62].

Data from Figure 5 are consistent with a CD25-mediated consumption of dendritic cell IL-2 by Tregs. These data provide compelling *in vitro* evidence in favor of the dendritic cell as the important source of IL-2.

### Dendritic cell IL-2 regulates Treg suppressive function *in vitro*

Darrasse-Jèze et al. showed that ablation of dendritic cells *in vivo* caused progressive loss of Tregs and of Foxp3 expression in Tregs as well as the development of autoimmunity [66]. Though the authors speculated that the mechanism by which dendritic cells sustain Treg homeostasis involves DC expression of MHC class II, the finding that *in vivo* the DC levels are proportional to the level of Foxp3 expression in Tregs [66] is consistent with the model that Treg functional fitness is maintained by IL-2-secreting dendritic cells. Under some experimental conditions, other DC cytokines (e.g., IL-15) can support Treg proliferation [67]. Unlike Treg proliferation, Treg function depends more exclusively on IL-2. Wuest et al. recently compared the *in vitro* suppressor function of Tregs that were pre-activated with different common, gamma-chain family cytokines: IL-2, IL-7, IL-15, and IL-21. Although IL-2, IL-7, and IL-15 each triggered STAT5 phosphorylation and upregulated Foxp3 in Tregs, only Tregs that were incubated with IL-2 showed potent inhibitory function [42]. If Tregs require IL-2 for their suppressor function, then the finding that wild-type Tregs effectively suppress IL-2-deficient T-cells [45] lends credence to a non-T-cell source of IL-2, such as dendritic cells.

Natural Tregs are known to express high levels of IL-2 receptor alpha chain (CD25) [68]. Figure 7 clearly demonstrates that Tregs require DC IL-2 to upregulate CD25, and Foxp3 levels are maintained at a higher level when Tregs are cultured in the presence of IL-2-sufficient dendritic cells. Tregs failed to upregulate CD25 upon stimulation with IL-2^−/−^ dendritic cells, ruling out autocrine IL-2 signaling from the Treg as the source driving CD25 expression. Figures 8 and 9 clearly demonstrate that Tregs require dendritic cell IL-2 to exhibit suppressor function. Tregs failed to suppress wild-type CD4^+^ Teff cells when IL-2^−/−^ dendritic cells are substituted for wild-type dendritic cells in Figure 9. Moreover, data presented in Figure 9 rule out contaminating CD4^+^ Teff cells as the paracrine source of IL-2 in our *in vitro* system. Of note, although CD4^+^ Teff cells secreted IL-2 in cultures with IL-2^−/−^ DCs, this IL-2 did not appear to be sufficient for Treg function. Instead, exogenous IL-2 was needed to restore the suppressor function of Treg (Figure 9E&F). Our data herein identify the dendritic cell as the necessary supplier of IL-2 to manifest Treg suppressor function.

The Tregs in all of our experiments were stimulated through their TCR, either with anti-CD3, OVA peptide, or alloantigen. Therefore we have not formally tested whether IL-2 from DCs can impart Treg suppressive function independent of TCR signaling. Although IL-2 stimulation of CD25 promoter activity has been shown without TCR signaling [52], it seems implausible that Tregs would gain suppressor function in the absence of TCR activation [69].

The ability of IL-2-producing DCs to equip Tregs for suppressive function *in vivo* remains to be determined. Our results do not rule out the possibility that IL-2-producing BMDCs may be an artifact of the *in vitro* culture conditions for generating BMDCs. However, that we find IL-2-producing DCs in freshly isolated spleen (Figures 2C and 3A) lends credibility to the existence of such cells *in vivo*. Furthermore, reports by Sgouroudis et al. that LPS-matured BMDCs secrete IL-2 enabling them to expand Treg cells *in vivo* [46] add promise that our *in vitro* findings will have *in vivo* significance.

### Delivery of essential IL-2 to Tregs facilitated by DC-Treg cell-cell contact and CD25

IL-2-producing dendritic cells showed a preference for conjugate formation with Treg cells compared with CD4^+^ Teff cells (Figure 4). Onishi et al. previously noted that compared with CD4^+^ Teff cells, Tregs are much more efficient in forming conjugates with DCs *in vitro* and outcompete Teff cells in this process in mixed co-cultures [70]. Tang et al. reported that Treg cells form lasting and stable conjugates with CD11c^+^ DCs in pancreatic draining lymph node of NOD mice following priming with autoantigen [51]. Importantly, that Tregs rarely encounter Teff but readily engage antigen bearing dendritic cells prior to the inhibition of Teff activation by dendritic cells, places the dendritic cells at the center of Treg cell function in vivo [51]. It is intriguing that the most IL-2-producing DCs are contained within the Treg-conjugated DCs (Figure 4). Perhaps Treg and IL-2-producing cells express unique sets of chemokines and chemokine receptors that promote special chemoattraction between these two relatively sparse populations, such that IL-2-producing dendritic cells and Tregs seek out each other. Indeed, unique chemokine receptor and ligands utilized between DCs and Tregs have been reported [71].

Because IL-2 transcription is preserved in DCs in conjugate formation with Tregs, yet DC secretion of IL-2 onto ELISPOT plates is not seen, we speculate that IL-2 secretion is redirected towards Tregs in contact with the DCs. We explored this possibility using genetically-modified dendritic cells that constitutively secrete an IL-2-mCherry fusion protein. The imaging and flow cytometry studies in Figure 6 confirmed that DCs preferentially deliver IL-2 to Treg cells and not Teff cells. Epifluorescence microscopy of the DC-Treg conjugates showed IL-2mCherry inside both DCs and Tregs in a vesicular-like distribution pattern (Figure 6B&C). The IL-2mCherry distribution pattern in the Tregs is consistent with receptor-mediated IL-2 cytokine endocytosis and post-endocytosis receptor-ligand complex processing [72], [73]. Moreover, contact between DCs and Tregs facilitates IL-2 uptake by Tregs (Figure 6D). This process depends on expression of CD25 on the Treg, because antibody against CD25 ablated the transfer of IL-2mCherry from DCs to Tregs.

Our data suggest that Tregs are uniquely capable of uptake of IL-2 from dendritic cells and utilize a cell-cell contact to facilitate this process. Altogether these data expand on the current knowledge of IL-2 requirement for Treg fitness and function and document a crucial role for cell-cell contact with dendritic cells and CD25 on Tregs for IL-2 delivery to Tregs. It is known that Tregs require lower concentrations of IL-2 than Teff cells for in vivo expansion [38]. This may be accomplished in part by a subset of dendritic cells with the ability to spatially control the concentration and target of IL-2 secretion.

### Next steps towards immunotherapeutic applications

To build on this understanding, future experiments are needed to determine the phenotype(s) of IL-2-producing dendritic cells and why these cells prefer conjugate formation with the Tregs. A next step to regulate the supply of IL-2 to Tregs will be to elucidate the mechanism that sustains DC transcription of IL-2. Furthermore, potential clinical applications of these findings will depend on whether IL-2-producing DCs regulate Treg function *in vivo* as well as whether similar DCs exist in humans to control Treg function. *In vivo* studies by Tang et al. support this line of investigation; they found Treg-DC contact preceded the inhibition of Teff cell activation by dendritic cells [51]. Additional studies utilizing DC-lineage-specific IL-2 knock-out and knock-in mice should address the *in vivo* relevance of our findings.

Even so, our *in vitro* findings warrant the immediate consideration of a few practical applications. To our knowledge immunotherapy protocols utilizing dendritic cells that have been manipulated *ex vivo* to become immunogenic or tolerogenic have not addressed the potential of IL-2-producing DCs to activate immunosuppression by Tregs [74]. We recommend that dendritic cells used in immunotherapy for cancer (or infectious diseases) be routinely screened for IL-2-producing DCs because inadvertent delivery of IL-2-producing DCs may undermine the effectiveness of DC-based therapy by promoting Treg suppressor function. Secondly, because we were able to increase Foxp3 message by co-culturing Tregs with IL-2 transfected DCs, we suggest that this methodology may offer an alternative approach to preferentially expand Tregs over Teff cells *ex vivo.*

### Summary

We show that a subset of dendritic cells constitutively secretes IL-2. We show this DC-produced IL-2 is delivered to and consumed by natural Tregs *in vitro*. We also show a juxtacrine mechanism enables the preferential delivery of IL-2 to Tregs, but not to Teff cells. Furthermore, we show that dendritic cell IL-2 is essential for acquisition of Treg suppressor function *in vitro*. These discoveries expand our knowledge of IL-2 requirement for Treg fitness and function and may help inform future experiments aimed at designing interventions to enhance or inhibit Treg function.

## Materials and Methods

### Mice and cell lines

C57BL/6, C.CgFoxp3tm2Tch/J, BALB/cJ mice, DO11.10 TCR transgenic breeding pairs and B6.129P2-Il2*^tm1Hor^*/J heterozygous breeding pairs were purchased from The Jackson Laboratory (Bar Harbor, ME) and bred at The University of Iowa Animal Care Facilities. Wild-type IL-2 sufficient mice (WT) and homozygous IL-2^−/−^ mice were identified from litters by genotyping tail DNA. The transgenic DO11.10 TCR recognizes OVA peptide 323–339 with H-2^d^ [75] and the alloantigen H-2^b^ in the absence of OVA peptide [76], [77]. C57Bl/6-Tg-IL2p8-4 transgenic mice were kindly provided by Drs. M. Yui and E. Rothenberg, California Institute of Technology [78]. C57BL/6-Tg-IL2p8-4 mice have 3 copies of the IL2p8-EGFP transgene and have been useful for tracking IL-2 positive cells through the expression of IL-2 promoter-driven EGFP [79]. All studies were conducted using protocols reviewed and approved #0591101, 0890902 by the Veterans Affairs and The University of Iowa Institutional Animal Care and Use Committee and in accordance with the Guide to the Care and Use of Laboratory Animals and the Animal Welfare Regulations (CFR, Title 9, Chapter 1, Subchapter A).

### Dendritic cell and CD4^+^ cell subset purification

Dendritic cells were purified from single-cell suspensions of splenocytes or BMDC cultures from indicated mice by one or two passes through AutoMACS using the Pan DC isolation kit (Miltenyi Biotec, Auburn, CA, USA) according to manufacturer's specifications. Enriched splenic DCs and BMDCs were blocked with anti-CD16/CD32 then stained with CD11c-APC (both eBioscience, San Diego, CA, USA) and analyzed for CD11c^+^ by flow cytometer. After 2 passes DCs purity (CD11c^+^) was 97–99%. For BMDCs, bone marrow was harvested from mouse tibias and femurs and grown in complete DMEM (Dulbecco's modified Eagle's medium) for 6 d with intermittent feeding every 48 h with 20 ng/mL GM-CSF (Miltenyi Biotec).

CD4^+^CD25^+^ (Treg) and CD4^+^CD25^−^ (Teff) populations were obtained from spleen cell suspensions from indicated mice using AutoMACS and the mouse Treg isolation kit (Miltenyi Biotec) according to manufacturer's specifications. FACS analysis of the subset populations showed CD4^+^CD25^+^ purity >97–98%.

### Dendritic production of IL-2

To detect intracytoplasmic IL-2 protein in dendritic cells, purified C57BL/6 or WT BMDCs were cultured 5 h with golgistop (eBioscience) followed with straining with Live/Dead Fixable Violet dead cell stain kit for 405 nm excitation (Invitrogen, Life Technologies). Cells were surface stained with anti-CD11c-APC, CD8a-FITC, CD205-PE-Cy7 then intracellular stained with anti-IL-2-PE or isotype control Rat IgG2b-PE (all antibodies from eBioscience) and analyzed by flow cytometry.

To detect secreted IL-2 protein, purified spleen DCs or BMDCs (10^4^/well) from C57BL/6 were cultured directly in IL-2-coated ELISPOT plates. Whatman ELISPOT plates were pre-coated with IL-2 capture antibody JES6-IA12, 2 µg/ml (eBiosciences) for O/N in 4°C. Plates were blocked with 150 µl/well 1xPBS-BSA 5% for 45 min at room temperature (RT), conditioned with 150 µl/well complete DMEM for 45 min at RT and then washed 4 times with 1XPBS before use. Dendritic cells were then cultured in ELISPOT plates for 24 h at 37°C in complete DMEM media, or in media supplemented with LPS (E Coli strain 0127:B8, 1 µg/ml, Sigma-Aldrich, St. Louis, MO, USA), CpG-A or CpG-B (each 1 µg/ml and kindly provided by Dr. Petar Lenert, The University of Iowa). In some experiments DO11.10 Tregs were added 1∶1 with C57BL/6 splenic DCs and BMDCs with or without anti-CD25 (PC61) or isotype control antibody Rat IgG1 (both BD Biosciences, San Diego, CA, USA), final concentration 3–30 µg/ml. IL-2 ELISPOT plates were thoroughly washed with 1xPBS 24 h later and IL-2 spots were detected using JES6-5H, 100 µl/well (eBioscience) for O/N at 4° C, then goat anti-rat Biotin-HRP (Sigma-Alrich) for O/N at 4°C, then AEC (3-amino-9-ethylcarbazole, Sigma-Aldrich) substrate as described [77]. Spots were counted and analyzed with the ImmunoSpot analyzer (Cellular Technology Limited, Shaker Heights, OH, USA).

To detect IL-2 message, total RNA was isolated (RNeasy, Qiagen, Valencia, CA, USA) from C57BL/6 splenic DCs or BMDCs and cDNA was synthesized from 100 ng RNA/sample using Invitrogen SuperScript First strand synthesis system. IL-2 transcription was measured by RT-qPCR on triplicate samples using FAM tagged mouse IL-2 primers (Mm00434256_m1, Applied Biosystems) and ABI PRISM 7700 Sequence Detection System according to manufacturer's protocols. Samples were standardized for GAPDH VIC-TAMARA and quantification of the gene of interest is given by 2 – delta Ct, where delta Ct is obtained by calculating the difference between Ct of the gene of interest and GAPDH. In one experiment C57BLl/6 splenic DCs and DO11.10 Treg cells were cultured in separate tube or together at 1∶1 for 5 and 24 h. The Tregs and DCs from separate tubes were combined and cells were immediately harvested from cultures for detection of IL-2 message by RT-qPRC.

To detect IL-2 promoter activity in dendritic cells, C57BL/6 or WT BMDCs were transfected with the IL-2 promoter-driven EGFP reporter plasmid (pIL2P8.4EGFP, kindly provided by Dr. Ellen Rothenberg, California institute of Technology, Pasadena, CA, [78]), negative control plasmid pcDNA3.1/V5-His B (Invitrogen, Grand Island, NY, USA), and positive control plasmid pAcGFP-N1 (ClonTech Laboratories), using Lonza/AMAXA-Immature DC Nucleofection Kit (Allendale, NJ, USA) according to manufacturer's specifications, and 24–40 h later cells were stained with anti-CD11c-PE-Cy7 (eBioscience) and analyzed for GFP^+^/CD11c^+^ or EGFP^+^/CD11c^+^ double positive cells using a BD LSR flow cytometer and FlowJo software (Tree Star, Inc., Ashland, OR). Transfection efficiency was determined by the percentage of GFP^+^ DCs in BMDCs that were transfected with pAcGFP-N1 and ranged from 15–35% in all experiments. In some experiments C57BL/6 transfectants were stimulated with CpG-B (1 µg/ml) and/or co-cultured with DO11.10 Treg or DO11.10 CD4^+^CD25^−^ cells at a 10∶1 ratio of Treg:DC or Teff:DC, and cells were stained with anti-CD11c-APC, anti-Thy1.2-PE-Cy7, and anti-CD25-PE (all from eBioscience). Multiparameter gating was used to measure IL-2 promoter-driven EGFP activity in different conjugated and nonconjugated populations.

### Treg phenotype and function

DO11.10 Treg cells were cultured alone in the upper chamber of transwell plates and with WT or IL-2^−/−^ (B6 background) DCs in the lower chamber of transwell plates. Cells were harvested from upper and lower chambers and treated with Live/Dead Fixable Dead Cell Stain (Invitrogen, Life Technologies), blocked with anti-CD16/32 (eBioscience) and stained with anti-Thy1.2-APC/anti-CD25-PE or isotype control Ab Rat IgG2a-APC and Rat IgG1-PE (BD Biosciences), fixed and permeabilized using Foxp3 Staining Buffer Set (eBioscience), and stained with anti-Foxp3-FITC or isotype control Rat IgG2a-FITC (eBioscience). CD25 and Foxp3 were measured on gated live Thy1.2^+^ cells by flow cytometry.

To measure Treg suppression of CD4 T-cell proliferation, naïve conventional CD4+ T cells (CD4^+^CD25^−^CD62L^+^) from C57BL/6 mice were labeled with CFSE (1 µM, Sigma-Aldrich) and cultured for 4 d at 1×10^5^ cells/ml in 12×75 mm polystyrene tubes in RPMI complete media with anti-CD3ε (0.3 µg/ml, 145-2C11, eBioscience) along with purified DCs from WT or IL-2^−/−^ (B6 background) mice (2.6×10^4^ cells/ml), with or without purified C57BL/6 CD4^+^CD25^+^ Treg cells (2.6×10^4^ cells/ml). Cells were harvested, blocked with anti-CD16/CD32 then stained with anti-CD11c-APC and anti-CD4-APC-Alexa750 (all from eBioscience). Hoechst 33258 (Sigma-Aldrich) was added for live/dead discrimination. Proliferation of CFSE-labeled conventional CD4+ cells (Teff) was measured on BD-LSR II flow cytometer and analyzed using FlowJo software (Tree Star, Inc.). In other experiments DO11.10 Treg (10^4^ cells/ml) and CFSE-labeled Teff cells (10^5^ cells/ml) were cultured with 10^4^ allogeneic DCs from WT and IL-2^−/−^ (B6 background) mice with or without OVA peptide 323–339 (100 µM, Sigma-Aldrich) for 4 d in 12×75 mm polystyrene tubes in RPMI complete media and proliferation of Teff was measured as above. Proliferation was measured by flow cytometry, and the % of Treg suppression of Teff cell proliferation was calculated by:
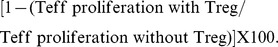



To measure Treg suppression of CD4 cell IL-2 production, 10^5^ DO11.10 CD4^+^CD25^−^ Teff were stimulated with 10^4^ allogeneic DCs from WT and IL-2^−/−^ (B6 background) mice overnight in 96-well plates with or without co-cultured 10^4^ DO11.10 CD4^+^CD25^+^ DO11.10 Tregs. In some experiments anti-CD25 (PC61) or isotype control Rat IgG1 (both BD Biosciences) was added (3 to 30 µg/ml). In other experiments recombinant murine IL-2 (eBioscience) was added (100 to 1000 U/ml). Cells were harvested, washed, then replated on IL-2 ELISPOT plates for 24 h. IL-2 spots were developed and quantified by ImmunoSpot (Cellular Technology Limited).

### Confocal microscopy

Foxp3-GFP Treg cells from C.CgFoxp3tm2Tch/J mice were stimulated with anti-CD3 (1 mg/ml) and unlabeled BALB/cJ splenic DCs for 6 h in presence or absence of unlabeled anti-CD25 antibody (PC61, 30 µg/ml). Live cell confocal imaging was performed using Leica TCS SP2 inverted confocal microscope (Leica Microsystems, Wetzlar, Germany). After identifying DC:Treg conjugates, anti-CD25-APC (7D4, Southern Biotec, Birmingham, AL, USA) was added to cultures and conjugates were imaged. Normarski differential interference contract (DIC), GFP and APC channels were collected sequentially at 0.3–0.5 micron z steps. LCS Lite software (Leica Microsystems) was used to quantitate CD25-APC intensity on Treg membrane not contacting and in contact with DC.

### Tracking and transfer of IL-2 between DCs and Tregs

To visualize the transfer of IL-2 between DCs and Tregs by microscopy, BMDCs were transfected with IL-2mCherry or mCherry-expressing plasmids using Lonza/AMAXA-Immature DC Nucleofection Kit according to manufacturer's specifications. In detail, engineered primers were used to delete the IL-2 gene stop codon, and the amplified product was sub-cloned into P-mCherry-N3 and related plasmids. These plasmids encoding IL-2 fluorescent fusion proteins were transfected into CD11c^+^ enriched, BMDCs by Amaxa nucleofection. We measured cytokine-induced proliferation of the HT-2 cell line (ATCC# CRL-1841) and CD25 up-regulation on Tregs to confirm that IL-2-mCherry and IL-2 had similar biological activity (not shown). Transfected DCs from BALB/c mice were washed and co-cultured for 24 h with purified and CFSE-labeled DO11.10 CD4^+^CD25^+^ Tregs or conventional CD4^+^ cells (CD4^+^CD25^−^, Teff) and OVA peptide 323–339 (100 µM, Sigma-Aldrich). Live-cell epifluorescence imaging was performed to track IL-2mCherry uptake by Tregs and Teff utilizing Olympus X-81 microscope with temperature and CO_2_ environmental controls and SlideBook software (Intelligent Imaging Innovations, Denver, CO, USA). To quantify the transfer of IL-2 from DCs to Tregs, BMDCs from IL-2^−/−^ B6 mice were transfected with IL-2mCherry or control mCherry plasmid, washed and co-cultured with purified DO11.10 CD4^+^CD25^+^ Tregs or DO11.10 conventional CD4^+^ cells (CD4^+^CD25^−^, Teff) in transwell culture plates with or without 30 µg/ml anti-CD25 (PC61, BD Biosciences) for 24 h at 37°C and 5% CO_2_ in triplicates. DCs were cultured in the lower chamber of the transwells. Tregs or Teff were cultured in both the lower and upper chambers. Anti-CD3 antibody (10 µg/ml) was added to the wells with Teff to further upregulate CD25 expression so that CD25 expression on Tregs and Teff were comparable. The DC:T cells ratio was between 1–1.6∶1. Cells were collected from top and bottom wells, blocked with anti-CD16/CD32 (eBioscience) and stained with anti-CD25-PE and Thy1.2-FITC or isotype control antibodies: rat IgG2b, κ-FITC, and rat IgG1, λ-PE (all eBioscience). Hoechst 33258 (Sigma-Aldrich) was added to discriminate live and dead cells. Cells were analyzed by BD LSR flow cytometer and FlowJo software (Tree Star, Inc.). Mean fluorescent intensity of mCherry and CD25 of Thy1.2^high^ gated cells was expressed as mean ± SEM.

### Statistical analysis

Data on the frequency of spontaneous IL-2 secreting BMDCs and Treg interference of DC secretion of IL-2 were analyzed using one-way, repeated measures ANOVA and Tukey's test for single-step, pairwise multiple comparison procedure. Mean fluorescent intensities of Foxp3 levels on Thy1.2-gated Tregs were compared using Student's *t*-test, or as specified in results, using SigmaStat software (San Jose, CA). Data for Treg suppression of MLR-IL-2 response were analyzed using the Holm-Sidak method of multiple pairwise comparisons or Tukey's test for pairwise comparison.

## References

[1] Sakaguchi S, Ono M, Setoguchi R, Yagi H, Hori S, et al (2006) Foxp3+ CD25+ CD4+ natural regulatory T cells in dominant self-tolerance and autoimmune disease. Immunol Rev 212: 8–27. 16903903 10.1111/j.0105-2896.2006.00427.x

[2] Wood KJ, Sakaguchi S (2003) Regulatory T cells in transplantation tolerance. Nat Rev Immunol 3: 199–210. 12658268 10.1038/nri1027

[3] Schulze Zur Wiesch J, Thomssen A, Hartjen P, Tóth I, Lehmann C, et al (2011) Comprehensive analysis of frequency and phenotype of T regulatory cells in HIV infection: CD39 expression of FoxP3+ T regulatory cells correlates with progressive disease. J Virol 85: 1287–1297. 21047964 10.1128/JVI.01758-10 PMC3020516

[4] Curiel TJ (2008) Regulatory T cells and treatment of cancer. Curr Opin Immunol 20: 241–246. 18508251 10.1016/j.coi.2008.04.008 PMC3319305

[5] Welters MJ, Kenter GG, de Vos van Steenwijk PJ, Löwik MJ, Berends-van der Meer DM, et al (2010) Success or failure of vaccination for HPV16-positive vulvar lesions correlates with kinetics and phenotype of induced T-cell responses. Proc Natl Acad Sci U S A 107: 11895–11899. 20547850 10.1073/pnas.1006500107 PMC2900675

[6] Riley JL, June CH, Blazar BR (2009) Human T regulatory cells as therapeutic agents: Take a billion or so of these and call me in the morning. Immunity 30: 656–665. 19464988 10.1016/j.immuni.2009.04.006 PMC2742482

[7] Shuntharalingam G, Perry MR, Ward S, Brett SJ, Castello-Cortes A, et al (2006) Cytokine storm in a phase 1 trial of the anti-CD28 monoclonal antibody TGN1412. N Engl J Med 355: 1018–1028. 16908486 10.1056/NEJMoa063842

[8] Brusko T, Bluestone J (2008) Clinical application of regulatory T cells for treatment of type 1 diabetes and transplantation. Eur J Immunol 38: 931–934. 18395864 10.1002/eji.200738108

[9] Putnam AL, Brusko TM, Lee MR, Liu W, Szot GL, et al (2009) Expansion of human regulatory T-cells from patients with type 1 diabetes. Diabetes 58: 652–662. 19074986 10.2337/db08-1168 PMC2646064

[10] Battaglia M, Roncarolo MG (2011) Immune intervention with T regulatory cells: past lessons and future perspectives for type 1 diabetes. Semin Immunol 23: 182–194. 21831659 10.1016/j.smim.2011.07.007

[11] Feng G, Wood KJ, Bushell A (2008) Interferon-gamma conditioning ex vivo generates CD25+CD62L+Foxp3+ regulatory T cells that prevent allograft rejection: potential avenues for cellular therapy. Transplantation 86: 578–589. 18724229 10.1097/TP.0b013e3181806a60

[12] Tang Q, Bluestone JA, Kang SM (2012) CD4(+)Foxp3(+) regulatory T cell therapy in transplantation. J Mol Cell Biol 4: 11–21. 22170955 10.1093/jmcb/mjr047 PMC3695644

[13] Edinger M, Hoffmann P (2011) Regulatory T cells in stem cell transplantation: strategies and first clinical experiences. Curr Opin Immunol 23: 679–684. 21802270 10.1016/j.coi.2011.06.006

[14] Bruno L, Merkenschlager M (2008) Directing T cell differentiation and function with small molecule inhibitors. Cell Cycle 7: 2296–2298. 18677111 10.4161/cc.6444

[15] Brusko TM, Putnam AL, Bluestone JA (2008) Human regulatory T cells: role in autoimmune disease and therapeutic opportunities. Immunol Rev 223: 371–390. 18613848 10.1111/j.1600-065X.2008.00637.x

[16] Ruter J, Barnett BG, Kryczek I, Brumlik MJ, Daniel BJ, et al (2009) Altering regulatory T cell function in cancer immunotherapy: a novel means to boost the efficacy of cancer vaccines. Front Biosci 14: 1761–1770. 10.2741/3338 19273160

[17] Welters MJ, Piersma SJ, van der Burg SH (2008) T-regulatory cells in tumour-specific vaccination strategies. Expert Opin Biol Ther 8: 1365–1379. 18694355 10.1517/14712598.8.9.1365

[18] Wei S, Kryczek I, Edwards RP, Zou L, Szeliga W, et al (2007) Interleukin-2 administration alters the CD4+FOXP3+ T-cell pool and tumor trafficking in patients with ovarian carcinoma. Cancer Res 67: 7487–7494. 17671219 10.1158/0008-5472.CAN-07-0565

[19] Berntsen A, Brimnes MK, thor Straten P, Svane IM (2010) Increase of circulating CD4+CD25highFoxp3+ regulatory T cells in patients with metastatic renal cell carcinoma during treatment with dendritic cell vaccination and low-dose interleukin-2. J Immunother 33: 425–434. 20386464 10.1097/CJI.0b013e3181cd870f

[20] Beyer M, Schumak B, Weihrauch MR, Andres B, Giese T, et al (2012) In vivo expansion of naive CD4+ CD25(high) FOXP3+ regulatory T cells in patients with colorectal carcinoma after IL-2 administration. PLoS One 7: e30422. 22276195 10.1371/journal.pone.0030422 PMC3262821

[21] Cheng G, Yu A, Malek TR (2011) T-cell tolerance and the multi-functional role of IL-2R signaling in T-regulatory cells. Immunol Rev 241: 63–76. 21488890 10.1111/j.1600-065X.2011.01004.x PMC3101713

[22] Barron L, Dooms H, Hoyer KK, Kuswanto W, Hofmann J, et al (2010) Cutting edge: mechanisms of IL-2-dependent maintenance of functional regulatory T cells. J Immunol 185: 6426–6430. 21037099 10.4049/jimmunol.0903940 PMC3059533

[23] Smith KA (2006) The structure of IL2 bound to the three chains of the IL2 receptor and how signaling occurs. Med Immunol 5: 3. 16907989 10.1186/1476-9433-5-3 PMC1562422

[24] Wang X, Rickert M, Garcia KC (2005) Structure of the quaternary complex of interleukin-2 with its alpha, beta, and gammac receptors. Science 310: 1159–1163. 16293754 10.1126/science.1117893

[25] Stauber DJ, Debler EW, Horton PA, Smith KA, Wilson IA (2006) Crystal structure of the IL-2 signaling complex: paradigm for a heterotrimeric cytokine receptor. Proc Natl Acad Sci U S A 103: 2788–2793. 16477002 10.1073/pnas.0511161103 PMC1413841

[26] Almeida AR, Legrand N, Papiernik M, Freitas AA (2002) Homeostasis of peripheral CD4+ T cells: IL-2R alpha and IL-2 shape a population of regulatory cells that controls CD4+ T cell numbers. J Immunol 169: 4850–4860. 12391195 10.4049/jimmunol.169.9.4850

[27] Malek TR, Yu A, Vincek V, Scibelli P, Kong L (2002) CD4 regulatory T cells prevent lethal autoimmunity in IL-2Rbeta-deficient mice. Implications for the nonredundant function of IL-2. Immunity 17: 167–178. 12196288 10.1016/s1074-7613(02)00367-9

[28] Papiernik M, de Moraes ML, Pontoux C, Vasseur F, Pénit C (1998) Regulatory CD4 T cells: expression of IL-2R alpha chain, resistance to clonal deletion and IL-2 dependency. Int Immunol 10: 371–378. 9620592 10.1093/intimm/10.4.371

[29] Soper DM, Kasprowicz DJ, Ziegler SF (2007) IL-2Rbeta links IL-2R signaling with Foxp3 expression. Eur J Immunol 37: 1817–1826. 17559173 10.1002/eji.200737101

[30] Sadlack B, Löhler J, Schorle H, Klebb G, Haber H, et al (1995) Generalized autoimmune disease in interleukin-2-deficient mice is triggered by an uncontrolled activation and proliferation of CD4+ T cells. Eur J Immunol 25: 3053–3059. 7489743 10.1002/eji.1830251111

[31] Suzuki H, Kündig TM, Furlonger C, Wakeham A, Timms E, et al (1995) Deregulated T cell activation and autoimmunity in mice lacking interleukin-2 receptor beta. Science 268: 1472–1476. 7770771 10.1126/science.7770771

[32] Willerford DM, Chen J, Ferry JA, Davidson L, Ma A, et al (1995) Interleukin-2 receptor alpha chain regulates the size and content of the peripheral lymphoid compartment. Immunity 3: 521–530. 7584142 10.1016/1074-7613(95)90180-9

[33] Bayer AL, Yu A, Adeegbe D, Malek TR (2005) Essential role for interleukin-2 for CD4(+)CD25(+) T regulatory cell development during the neonatal period. J Exp Med 201: 769–777. 15753210 10.1084/jem.20041179 PMC2212835

[34] Malek TR (2003) The main function of IL-2 is to promote the development of T regulatory cells. J Leukoc Biol 74: 961–965. 12960253 10.1189/jlb.0603272

[35] Furtado GC, Curotto de Lafaille MA, Kutchukhidze N, Lafaille JJ (2002) Interleukin 2 signaling is required for CD4(+) regulatory T cell function. J Exp Med 196: 851–857. 12235217 10.1084/jem.20020190 PMC2194060

[36] Yu A, Malek TR (2006) Selective availability of IL-2 is a major determinant controlling the production of CD4+CD25+Foxp3+ T regulatory cells. J Immunol 177: 5115–5121. 17015695 10.4049/jimmunol.177.8.5115

[37] Setoguchi R, Hori S, Takahashi T, Sakaguchi S (2005) Homeostatic maintenance of natural Foxp3(+) CD25(+) CD4(+) regulatory T cells by interleukin (IL)-2 and induction of autoimmune disease by IL-2 neutralization. J Exp Med 201: 723–735. 15753206 10.1084/jem.20041982 PMC2212841

[38] Tang Q, Adams JY, Penaranda C, Melli K, Piaggio E, et al (2008) Central role of defective interleukin-2 production in the triggering of islet autoimmune destruction. Immunity 28: 687–697. 18468463 10.1016/j.immuni.2008.03.016 PMC2394854

[39] Barron L, Dooms H, Hoyer KK, Kuswanto W, Hofmann J, et al (2010) Cutting edge: mechanisms of IL-2-dependent maintenance of functional regulatory T cells. J Immunol 185: 6426–6430. 21037099 10.4049/jimmunol.0903940 PMC3059533

[40] Thornton AM, Donovan EE, Piccirillo CA, Shevach EM (2004) Cutting edge: IL-2 is critically required for the in vitro activation of CD4+CD25+ T cell suppressor function. J Immunol 172: 6519–6523. 15153463 10.4049/jimmunol.172.11.6519

[41] de la Rosa M, Rutz S, Dorninger H, Scheffold A (2004) Interleukin-2 is essential for CD4+CD25+ regulatory T cell function. Eur J Immunol 34: 2480–2488. 15307180 10.1002/eji.200425274

[42] Wuest TY, Willette-Brown J, Durum SK, Hurwitz AA (2008) The influence of IL-2 family cytokines on activation and function of naturally occurring regulatory T cells. J Leukoc Biol 84: 973–980. 18653463 10.1189/jlb.1107778 PMC2538590

[43] Sakaguchi S, Sakaguchi N, Asano M, Itoh M, Toda M (1995) Immunologic self-tolerance maintained by activated T cells expressing IL-2 receptor alpha-chains (CD25). Breakdown of a single mechanism of self-tolerance causes various autoimmune diseases. J Immunol 155: 1151–1164. 7636184

[44] Fehérvari Z, Yamaguchi T, Sakaguchi S (2006) The dichotomous role of IL-2: tolerance versus immunity. Trends Immunol 27: 109–111. 16459146 10.1016/j.it.2006.01.005

[45] Wolf M, Schimpl A, Hünig T (2001) Control of T cell hyperactivation in IL-2-deficient mice by CD4(+)CD25(−) and CD4(+)CD25(+) T cells: evidence for two distinct regulatory mechanisms. Eur J Immunol 31: 1637–1645. 11385607 10.1002/1521-4141(200106)31:6<1637::aid-immu1637>3.0.co;2-t

[46] Sgouroudis E, Kornete M, Piccirillo CA (2011) IL-2 production by dendritic cells promotes Foxp3+ regulatory T-cell expansion in autoimmune-resistant NOD congenic mice. Autoimmunity 44: 406–414. 21244339 10.3109/08916934.2010.536795

[47] Guiducci C, Valzasina B, Dislich H, Colombo MP (2005) CD40/CD40L interaction regulates CD4+CD25+ T reg homeostasis through dendritic cell-produced IL-2. Eur J Immunol 35: 557–567. 15682445 10.1002/eji.200425810

[48] Zanoni I, Foti M, Ricciardi-Castagnoli P, Granucci F (2005) TLR-dependent activation stimuli associated with Th1 responses confer NK cell stimulatory capacity to mouse dendritic cells. J Immunol 175: 286–292. 15972660 10.4049/jimmunol.175.1.286

[49] Rogers NC, Slack EC, Edwards AD, Nolte MA, Schulz O, et al (2005) Syk-dependent cytokine induction by Dectin-1 reveals a novel pattern recognition pathway for C type lectins. Immunity 22: 510–517. 10.1016/j.immuni.2005.03.004 15845454

[50] Goodridge HS, Simmons RM, Underhill DM (2007) Dectin-1 stimulation by Candida albicans yeast or zymosan triggers NFAT activation in macrophages and dendritic cells. J Immunol 178: 3107–3115. 17312158 10.4049/jimmunol.178.5.3107

[51] Tang Q, Adams JY, Tooley AJ, Bi M, Fife BT, et al (2006) Visualizing regulatory T cell control of autoimmune responses in nonobese diabetic mice. Nat Immunol 7: 83–92. 16311599 10.1038/ni1289 PMC3057888

[52] Kim HP, Leonard WJ (2002) The basis for TCR-mediated regulation of the IL-2 receptor alpha chain gene: role of widely separated regulatory elements. EMBO J 21: 3051–3059. 12065418 10.1093/emboj/cdf321 PMC126074

[53] Murawski MR, Litherland SA, Clare-Salzler MJ, Davoodi-Semiromi A (2006) Upregulation of Foxp3 expression in mouse and human Treg is IL-2/STAT5 dependent: implications for the NOD STAT5B mutation in diabetes pathogenesis. Ann N Y Acad Sci 1079: 198–204. 17130555 10.1196/annals.1375.031

[54] Sojka DK, Hughson A, Sukiennicki TL, Fowell DJ (2005) Early kinetic window of target T cell susceptibility to CD25+ regulatory T cell activity. J Immunol 175: 7274–7280. 16301632 10.4049/jimmunol.175.11.7274

[55] Thornton AM, Shevach EM (1998) CD4+CD25+ immunoregulatory T cells suppress polyclonal T cell activation in vitro by inhibiting interleukin 2 production. J Exp Med 188: 287–296. 9670041 10.1084/jem.188.2.287 PMC2212461

[56] Granucci F, Vizzardelli C, Pavelka N, Feau S, Persico M, et al (2001) Inducible IL-2 production by dendritic cells revealed by global gene expression analysis. Nat Immunol 2: 882–888. 11526406 10.1038/ni0901-882

[57] Granucci F, Feau S, Angeli V, Trottein F, Ricciardi-Castagnoli P (2003) Early IL-2 production by mouse dendritic cells is the result of microbial-induced priming. J Immunol 170: 5075–5081. 12734352 10.4049/jimmunol.170.10.5075

[58] Sauma D, Michea P, Lennon-Duménil AM, Fierro A, Morales J, et al (2004) Interleukin-4 selectively inhibits interleukin-2 secretion by lipopolysaccharide-activated dendritic cells. Scand J Immunol 59: 183–189. 14871295 10.1111/j.0300-9475.2004.01380.x

[59] Marguti I, Yamamoto GL, da Costa TB, Rizzo LV, de Moraes LV (2009) Expansion of CD4+ CD25+ Foxp3+ T cells by bone marrow-derived dendritic cells. Immunology 127: 50–61. 18778287 10.1111/j.1365-2567.2008.02927.x PMC2678181

[60] Chen Y, Peng Z (2001) A sensitive in situ ELISA for quantitative measurements of cytokines and antibodies secreted by culture lymphocytes. J Immunoassay Immunochem 22: 353–369. 11816803 10.1081/ias-100107400

[61] Ekerfelt C, Ernerudh J, Jenmalm MC (2002) Detection of spontaneous and antigen-induced human interleukin-4 responses in vitro: comparison of ELISPOT, a novel ELISA and real-time RT-PCR. J Immunol Methods 260: 55–67. 11792376 10.1016/s0022-1759(01)00520-8

[62] Brinster C, Shevach EM (2005) Bone marrow-derived dendritic cells reverse the anergic state of CD4+CD25+ T cells without reversing their suppressive function. J Immunol 175: 7332–7340. 16301639 10.4049/jimmunol.175.11.7332

[63] Fehérvári Z, Sakaguchi S (2004) Control of Foxp3+ CD25+CD4+ regulatory cell activation and function by dendritic cells. Int Immunol 16: 1769–1780. 15520045 10.1093/intimm/dxh178

[64] Yamazaki S, Iyoda T, Tarbell K, Olson K, Velinzon K, et al (2003) Direct expansion of functional CD25+ CD4+ regulatory T cells by antigen-processing dendritic cells. J Exp Med 198: 235–247. 12874257 10.1084/jem.20030422 PMC2194081

[65] Suffner J, Hochweller K, Kühnle MC, Li X, Kroczek RA, et al (2010) Dendritic cells support homeostatic expansion of Foxp3+ regulatory T cells in Foxp3.LuciDTR mice. J Immunol 184: 1810–1820. 20083650 10.4049/jimmunol.0902420

[66] Darrasse-Jèze G, Deroubaix S, Mouquet H, Victora GD, Eisenreich T, et al (2009) Feedback control of regulatory T cell homeostasis by dendritic cells in vivo. J Exp Med 206: 1853–1862. 19667061 10.1084/jem.20090746 PMC2737156

[67] Koenen HJ, Fasse E, Joosten I (2003) IL-15 and cognate antigen successfully expand de novo-induced human antigen-specific regulatory CD4+ T cells that require antigen-specific activation for suppression. J Immunol 171: 6431–6441. 14662842 10.4049/jimmunol.171.12.6431

[68] Piccirillo CA, Thornton AM (2004) Cornerstone of peripheral tolerance: naturally occurring CD4+CD25+ regulatory T cells. Trends Immunol 25: 374–380. 15207505 10.1016/j.it.2004.04.009

[69] Ohkura N, Sakaguchi S (2010) Regulatory T cells: roles of T cell receptor for their development and function. Semin Immunopathol 32: 95–106. 20179931 10.1007/s00281-010-0200-5

[70] Onishi Y, Fehervari Z, Yamaguchi T, Sakaguchi S (2008) Foxp3+ natural regulatory T cells preferentially form aggregates on dendritic cells in vitro and actively inhibit their maturation. Proc Natl Acad Sci U S A 105: 10113–10118. 18635688 10.1073/pnas.0711106105 PMC2481354

[71] Muthuswamy R, Urban J, Lee JJ, Reinhart TA, Bartlett D, et al (2008) Ability of mature dendritic cells to interact with regulatory T cells is imprinted during maturation. Cancer Res 68: 5972–5978. 18632653 10.1158/0008-5472.CAN-07-6818 PMC2905229

[72] Hémar A, Subtil A, Lieb M, Morelon E, Hellio R, et al (1995) Endocytosis of interleukin 2 receptors in human T lymphocytes: distinct intracellular localization and fate of the receptor alpha, beta, and gamma chains. J Cell Biol 129: 55–64. 7698995 10.1083/jcb.129.1.55 PMC2120376

[73] Yu A, Malek TR (2001) The proteasome regulates receptor-mediated endocytosis of interleukin-2. J Biol Chem 276: 381–385. 11032838 10.1074/jbc.M007991200

[74] Kalantari T, Kamali-Sarvestani E, Ciric B, Karimi MH, Kalantari M, et al (2011) Generation of immunogenic and tolerogenic clinical-grade dendritic cells. Immunol Res 51: 153–160. 22105838 10.1007/s12026-011-8255-5 PMC3474330

[75] Murphy KM, Heimberger AB, Loh DY (1990) Induction by antigen of intrathymic apoptosis of CD4+CD8+TCRlo thymocytes in vivo. Science 250: 1720–1723. 2125367 10.1126/science.2125367

[76] Liu CP, Kappler JW, Marrack P (1996) Thymocytes can become mature T cells without passing through the CD4+ CD8+, double-positive stage. J Exp Med 184: 1619–1630. 8920852 10.1084/jem.184.5.1619 PMC2192895

[77] Rigby SM, Rouse T, Field EH (2003) Total lymphoid irradiation nonmyeloablative preconditioning enriches for IL-4-producing CD4+-TNK cells and skews differentiation of immunocompetent donor CD4+ cells. Blood 101: 2024–2032. 12406908 10.1182/blood-2002-05-1513

[78] Yui MA, Hernández-Hoyos G, Rothenberg EV (2001) A new regulatory region of the IL-2 locus that confers position-independent transgene expression. J Immunol 166: 1730–1739. 11160218 10.4049/jimmunol.166.3.1730

[79] Yui MA, Sharp LL, Havran WL, Rothenberg EV (2004) Preferential activation of an IL-2 regulatory sequence transgene in TCR gamma delta and NKT cells: subset-specific differences in IL-2 regulation. J Immunol 172: 4691–4699. 15067044 10.4049/jimmunol.172.8.4691

